# Short exposure to tranexamic acid does not affect, in vitro, the viability of human chondrocytes

**DOI:** 10.1186/s40001-019-0373-x

**Published:** 2019-02-22

**Authors:** Remo Goderecci, Ilaria Giusti, Stefano Necozione, Benedetta Cinque, Sandra D’Ascenzo, Vincenza Dolo, Vittorio Calvisi

**Affiliations:** Department of Life, Health and Environmental Sciences, University of L’Aquila, Piazzale Salvatore Tommasi 1, Blocco 11, 67100 L’Aquila, Italy

**Keywords:** Human chondrocytes, Tranexamic acid, Cartilage cells, Cytotoxicity, Orthopedic surgery

## Abstract

**Background:**

Only few studies have investigated the effect of topical application of tranexamic acid (TXA) on “minimally” invasive joint surgical procedures in which articular cartilage is preserved; for this reason, actually many surgeons avoid the use of topical TXA even if the disadvantage related to a blood loss can occur. The aim of this study was to evaluate the cytotoxicity, on human chondrocytes, of TXA at different concentrations and times of exposure and the mechanisms of cell death.

**Methods:**

Experiments were carried out on isolated human chondrocytes harvested from eight patients who underwent total knee replacement. Cell viability was determined using XTT assay and was assessed at 0, 24 and 48 h intervals after a 10-min-long treatment, followed by thorough washes, or at 24 and 48 h of treatment at TXA concentrations of 20, 50, 70 and 100 mg/ml. Cell cycle alterations and occurrence of cell death for apoptosis or necrosis were assessed by cytofluorimetry. Data were analyzed using Proc Mixed Procedure; LSMEANS was used to compare multiple group means with Tukey’s honestly significant difference test.

**Results:**

A significant correlation between the controlled for factors (type of treatment, time and concentration) was found in the performed experiment. No significant effect on cell viability was observed when the TXA exposure was limited to 10 min, while for increased exposure, 24 and 48 h, a remarkable reduction was found; cell death occurred by apoptosis and was already appreciable after 24 h, reaching a statistical significance after the 48-h-long treatment.

**Conclusion:**

A prolonged exposure to TXA may cause cartilage damage, thus its topical application can be expanded also to clinical scenarios that include retention of native cartilage chondrocytes, only if it is limited to few minutes and used at concentrations of 70 mg/ml or less.

## Background

The topical administration of tranexamic acid (TXA) has emerged as an effective way to decrease blood loss and the subsequent need for transfusion following total joint arthroplasties, such as total hip replacement (THR) and total knee replacement (TKR) [1, 2]. Although the intravenous administration of TXA has been accepted as a safe method, concerns exist regarding its possible systemic effects and, particularly, thromboembolic events [3, 4]; for this and other reasons (such as increased concentration at the operative site and a better surgeon control), orthopedic surgeons have increased their interest in the topical use of TXA [5].

Unlike THR and TKR, for which the in situ application of TXA is widely documented and accepted, to date, only few studies have investigated the effect of the topical application of TXA on “minimally” invasive surgical procedures (ligament reconstructions, arthroscopies, hemiarthroplasties and unicompartmental knee replacements), whose aim is the retention and preservation of the native cartilage. In the latter cases, the application of TXA in the local site of surgery could effectively reduce blood loss [6–8].

Since only few studies have investigated the effect of TXA on cartilage, without any definitive answer about cytotoxicity, actually many surgeons avoid the use of topical TXA when native cartilage is maintained, even where disadvantages associated with blood loss can occur [9, 10].

Hypothesizing that TXA application could affect chondrocytes biology, the aim of this study was to evaluate, in vitro, whether TXA has a cytotoxic effect on human chondrocytes and, if positive, to verify a dose and time dependence.

## Methods

### Ethics statement

The protocols employed in this study and the use of human tissues were approved by the Internal Review Board of University of L’Aquila (Protocol Number 10/2018). All patients were informed and provided written informed consent to participate in the study.

### Cell isolation and culture

Primary chondrocyte cultures were obtained from eight patients (four males and four females; age range 60–82, mean age 73, 25 years) affected by knee osteoarthritis (without any inflammatory and any other medical diseases) undergoing TKR. The specimens were obtained from osteochondral resections of distal femur and proximal tibia by saw, performed as provided for by the conventional TKR surgical procedure; since in all patients, the Kellgren–Lawrence grade was 4 and osteoarthritis had progressed at the medial side of knee, the study was conducted using relatively intact cartilage from the lateral femoral and tibial condyles.

For cell isolation, the cartilage was removed from the subchondral bone using a sterile sharp scalpel and slivers were diced to improve the subsequent enzyme action: overnight digestion in 1 mg/ml Collagenase Type II (Gibco-ThermoFisher, Waltham, Massachusetts, USA) in DMEM (EuroClone S.p.A., Pero (MI), Italy) supplemented with 10% fetal bovine serum (FBS-EuroClone). After digestion, the cell suspension was filtered through Falcon™ cell strainers (mesh size 70 µm; FisherScientific, ThermoFisher) to remove undigested tissue and centrifuged (450*g*, 10′, RT). Then, cells were resuspended in complete medium (DMEM, 1× penicillin/streptomycin, 2 mM l-glutamine (both from EuroClone), 10 mM Hepes and 2,5 µg/ml amphotericin B (both from Sigma-Aldrich, St. Louis, MO, USA), 10% FBS), seeded (25,000 cells/cm^2^), incubated at 37 °C, 5% CO_2_ and allowed to adhere and spread for a few days. All experiments were conducted on passage 1 chondrocytes to minimize the extent of dedifferentiation over subcultures.

### Cell viability

Cell viability was determined using an XTT assay (Sigma-Aldrich). Living cells metabolically reduce XTT to produce a colored water-soluble formazan; its optical density is directly proportional to the number of viable cells.

Briefly, 8000 cells/well were seeded onto a 96-well plate, incubated in complete medium at 37 °C, 5% CO_2_ for 96 h to enable cell adhesion and spreading, and then treated with TXA.

We used a water-dissolved TXA (W-TXA) (to generate a formulation similar to the commercially available pharmaceutical composition) or a complete medium-dissolved TXA (M-TXA) formulation. Since the water used as TXA solvent could itself affect cell viability, the cells were treated with water for injections (WFI) (Acqua per soluzioni iniettabili, Industria Farmaceutica Galenica Senese s.r.l. Monteroni d’Arbia, Siena, Italy) at a volume similar to that used for treatment (as control).

Cells were incubated for 10 min (10′) at 37 °C, 5% CO_2_ with WFI, W-TXA or M-TXA, diluted in complete medium to reach the desired concentrations (20, 50, 70, 100 mg/ml) and then extensively washed in complete medium; the effects on cell viability were observed immediately after washing (10′/0 h) and after 24 or 48 h (10′/24 h and 10′/48 h) culture in complete medium. Cells were also incubated with WFI, W-TXA or M-TXA for 24 or 48 h (24 h and 48 h).

At the end of each period, the XTT assay was performed and, after 4 h, the optical density (OD) was evaluated at 450 nm by an ELISA reader.

All experiments were performed in triplicate and repeated eight times.

### Cell cycle profile and apoptosis analysis by flow cytometer

For cell cycle, chondrocytes were treated with 100 mg/ml M-TXA as described above. Once treatments ended, untreated and treated cells were detached with Tryple Express (Gibco), washed with PBS, collected by centrifugation (450*g*, 10 min, 4 °C), counted and fixed in cold 70% ethanol solution (EtOH) in PBS, with gentle mixing at 4 °C for 30 min. Fixed cells (10^6^ cells/ml) were transferred to plastic BD tubes (Becton–Dickinson), washed twice with ice-cold PBS and stained with a solution containing 50 μg/ml PI, 0.1% Nonidet-P40 and RNase A (6 μg/10^6^ cell) (all from Sigma-Aldrich) in the dark (30 min, 4 °C). Data from 10,000 events per sample were collected and analyzed using FACS Calibur instrument (BD Instruments Inc.) equipped with cell cycle analysis software (Modfit LT for Mac V3.0).

To ascertain whether the chondrocytes’ viability reduction induced by TXA is associated with the induction of apoptosis or necrosis, the chondrocytes were treated with M-TXA 100 mg/ml for 24 and 48 h. Then, the apoptosis and necrosis were measured by flow cytometry analysis using the Annexin V Detection kit (Invitrogen by Thermo Fisher Scientific—Waltham, MA, USA), which allows to detect and quantify live, early/late apoptotic and necrotic cells. Flow cytometric procedures were carried out according to the instructions provided by the manufacturer. Prior to flow cytometric analysis, 200 µl of 1× binding buffer was added and 10,000 cells from each treated group were acquired by flow cytometry using a FACS Calibur (BD Instruments Inc.) equipped with a single laser emitting excitation light at 488 nm. Data analysis was performed in a Becton–Dickinson FACS Calibur flow cytometer using CellQuest software program (BD Instruments Inc.).

### Statistical analysis

The study was based on an estimated sample size of 24 experiments, with a 1:1:1 ratio for the three types of treatment (WFI, W-TXA, M-TXA), which was calculated to be adequate to achieve 90% power to detect a large effect size (Cohen’s *f*: 0.40) with 2 df and an *α* of 0.05 on the OD percentage values between the three types of treatments. Statistical power was calculated using G*POWER Version 3.1.9.2.

The data obtained from the calculation of OD are expressed as the percentage ± standard deviations compared to the control experiment (conventionally set to 100%) and were analyzed using Proc Mixed Procedure with subjects (experiments) treated as a random factor and treatment, concentration and moment as fixed factors. LSMEANS was used to compare multiple group means with Tukey’s honestly significant difference (HSD) test. Analysis was performed on variables logarithmically transformed to enhance symmetry of measures. Differences were considered significant when *p* ≤ 0.05.

Statistical analysis was carried out using SAS System version 9.5.

## Results

### Effects in vitro of TXA on chondrocyte viability

**Fig. 1:**
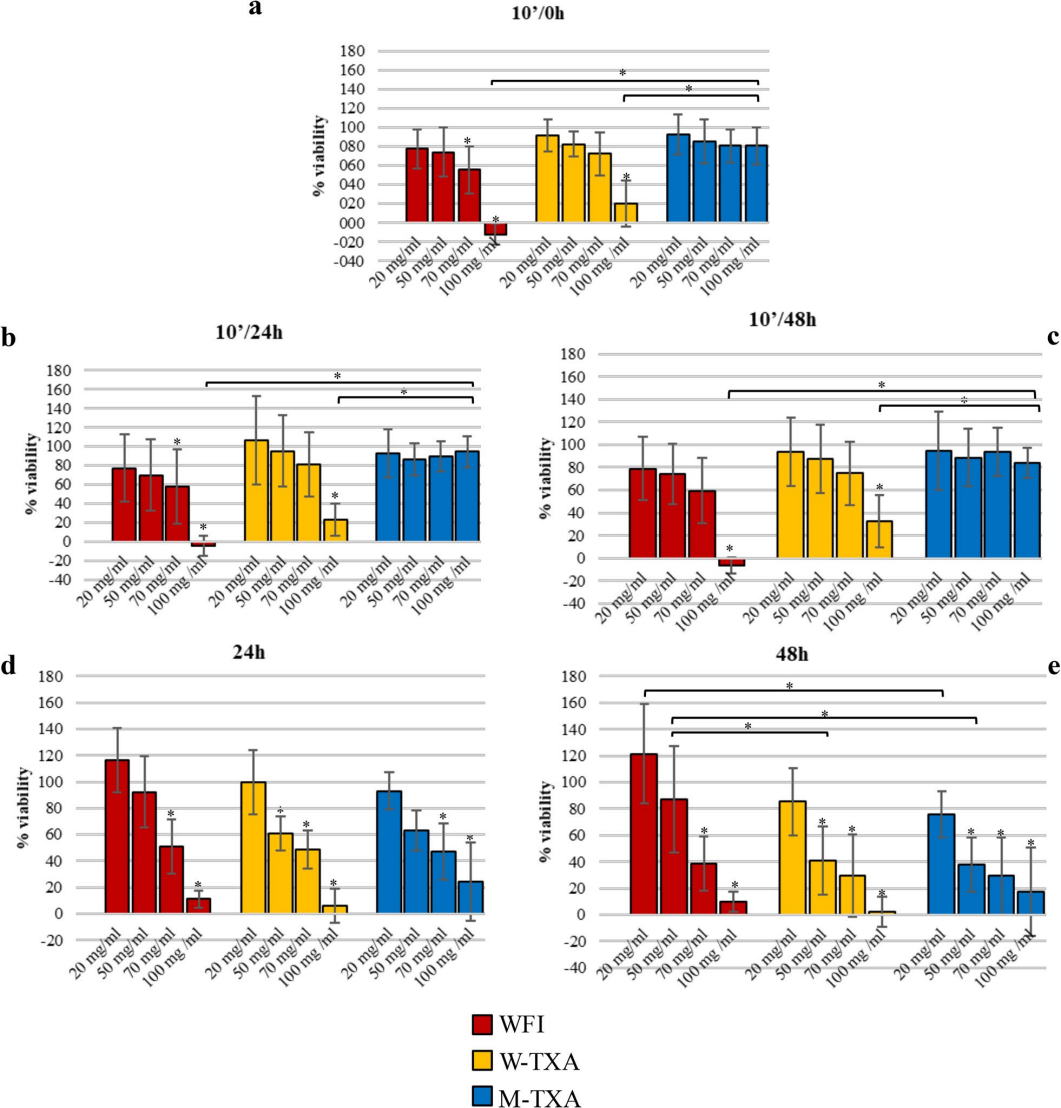
Tranexamic acid effects on chondrocytes viability. Cells were treated with W-TXA or M-TXA for 10 min and then extensively washed; effects on cell viability were observed soon after treatment—10′/0 h (**a**) or after 24 h—10′/24 h (**b**) or 48 h—10′/48 h (**c**) culture in complete medium. Cells were also incubated with W-TXA or M-TXA for 24 h—24 h (**d**) or 48 h—48 h (**e**). Graphs also report viability tests conducted using the same volumes of water (WFI) used in the experiments with W-TXA. Data originated from eight cell cultures, all analyzed in triplicate, and are expressed as % of viability (mean ± SD); in all graphs, the value 100% was conventionally assigned to the viability of untreated cells. The horizontal bars reported on the graphs highlight the significant differences between treatments for each single moment and the asterisks reported on each single bar represent a statistically significant difference of treated cells compared to untreated ones (*p ≤ 0.05 for Tukey’s test)

The W-TXA and the corresponding volume of WFI affected the viability at all times in a dose-dependent manner: when cells were washed after the 10-min-long treatment, the highest dose showed a severe effect on cell viability (Fig. 1a–c). When no washing was performed, both the 70 and 100 mg/ml doses for WFI and 50, 70 and 100 mg/ml for W-TXA strongly affected chondrocyte viability at 24 h and 48 h (Fig. 1d, e). The M-TXA formulation resembled the dose-dependent response of the W-TXA form in 24 h and 48 h treatments (Fig. 1d, e), while it did not show any detrimental effects when TXA was extensively washed after the 10-min treatment (Fig. 1a, c).

Graphs in Fig. 1 report the average response to the different treatments of the eight cellular preparations and it is representative of the tendency of viability according to moments (10′/0 h, 10′/24 h, 10′/48 h, 24 h, 48 h) and concentrations (20, 50, 70, 100 mg/ml); for greater clarity, the same data are reported in Table 1. Table 2 reports the significant differences between moments for each treatment.

**Table 1:** Percentage of viability for each treatment at all concentrations and moments

	20 mg/ml	50 mg/ml	70 mg/ml	100 mg/ml
10′/0 h				
Untreated % viability	100 ± 0.00	100 ± 0.00	100 ± 0.00	100 ± 0.00
WFI % viability	77.75 ± 20.42	74.10 ± 25.56	55.45 ± 25.03	− 11.95 ± 10.92
W-TXA % viability	91.77 ± 16.76	82.56 ± 12.92	72.21 ± 22.90	20.04 ± 24.36
M-TXA % viability	92.58 ± 21.31	85.34 ± 23.30	80.66 ± 17.37	80.62 ± 19.54
10′/24 h				
Untreated % viability	100 ± 0.00	100 ± 0.00	100 ± 0.00	100 ± 0.00
WFI % viability	77.46 ± 35.21	70.12 ± 36.97	57.67 ± 38.81	− 4.14 ± 10.54
W-TXA % viability	106.48 ± 46.19	95.36 ± 37.60	81.53 ± 33.80	22.88 ± 16.75
M-TXA % viability	92.45 ± 25.20	86.79 ± 16.73	89.49 ± 15.91	94.38 ± 15.99
10′/48 h				
Untreated % viability	100 ± 0.00	100 ± 0.00	100 ± 0.00	100 ± 0.00
WFI % viability	78.78 ± 28.05	74.40 ± 26.51	59.43 ± 28.64	− 6.32 ± 6.79
W-TXA % viability	94.02 ± 30.07	87.85 ± 30.19	74.73 ± 28.23	32.51 ± 23.24
M-TXA % viability	94.45 ± 34.62	88.82 ± 25.44	93.74 ± 21.35	84.15 ± 13.19
24 h				
Untreated % viability	100 ± 0.00	100 ± 0.00	100 ± 0.00	100 ± 0.00
WFI % viability	116.66 ± 24.37	92.40 ± 26.77	50.93 ± 20.52	11.06 ± 6.23
W-TXA % viability	99.33 ± 24.38	60.97 ± 12.99	48.93 ± 14.46	5.99 ± 12.91
M-TXA % viability	93.13 ± 14.37	63.38 ± 15.14	47.25 ± 21.51	24.34 ± 29.53
48 h				
Untreated % viability	100 ± 0.00	100 ± 0.00	100 ± 0.00	100 ± 0.00
WFI % viability	121.51 ± 37.41	87.10 ± 40.25	38.76 ± 20.37	9.95 ± 7.51
W-TXA % viability	85.22 ± 25.67	41.16 ± 25.81	29.35 ± 30.97	2.38 ± 11.48
M-TXA % viability	75.83 ± 17.39	38.07 ± 20.36	29.29 ± 29.23	17.41 ± 33.14

Average values of OD for each type of treatment and each concentration are expressed as percentage compared to the control experiment OD values (assumed as 100%). Untreated: control experiment; WFI: cells treated with water for injections; W-TXA: cells treated with water-dissolved tranexamic acid; M-TXA: cells treated with medium-dissolved tranexamic acid

**Table 2:** Statistical significance between moments

	Concentration	*p* ≤ 0.05
WFI	20 mg/ml	10′/0 h vs. 24 h
		10′/0 h vs. 48 h
		10′/24 h vs. 48 h
		10′/48 h vs. 48 h
W-TXA	50 mg/ml	10′/0 h vs. 48 h
		10′/24 h vs. 48 h
		10′/48 h vs. 48 h
	70 mg/ml	10′/0 h vs. 48 h
		10′/24 h vs. 48 h
		10′/48 h vs. 48 h
M-TXA	50 mg/ml	10′/0 h vs. 48 h
		10′/24 h vs. 48 h
		10′/48 h vs. 48 h
	70 mg/ml	10′/0 h vs. 48 h
		10′/24 h vs. 24 h
		10′/24 h vs. 48 h
		10′/48 h vs. 24 h
		10′/48 h vs. 48 h
	100 mg/ml	10′/0 h vs. 24 h
		10′/0 h vs. 48 h
		10′/24 h vs. 24 h
		10′/24 h vs. 48 h
		10′/48 h vs. 24 h
		10′/48 h vs. 48 h

The table reports only the statistically significant differences between moments (10′/0 h, 10′/24 h, 10′/48 h, 24 h, 48 h) for each treatment (WFI, W-TXA, M-TXA) (*p* ≤ 0.05 for Tukey’s test)

We found a significant interaction between the controlled for factors (treatments, moments, concentrations) (*p* = 0.020).

### Effects in vitro of TXA on apoptosis levels and cell cycle profile of chondrocytes

**Fig. 2:**
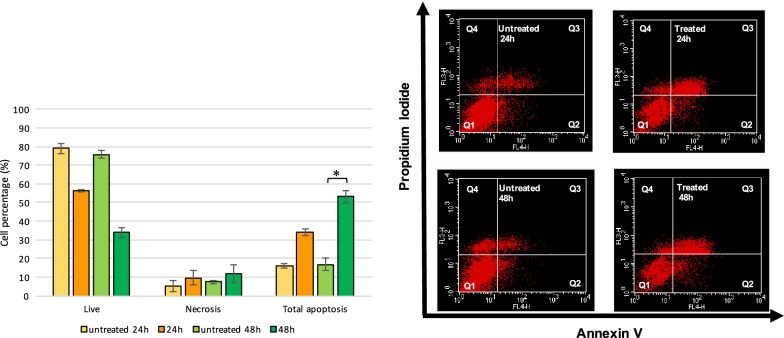
Effect of TXA on chondrocyte apoptosis level. On the left, the percentages of live, necrotic and apoptotic cells as assessed with Annexin V–APC/PI dual staining and expressed as cell percentage (mean ± SEM) are reported (*p < 0.05). On the right, the representative cytofluorimetric profiles of AnV/PI staining are also shown. In apoptotic cells, the externalization of membrane phosphatidylserine (PS), a crucial step of apoptosis induction, is identified by Annexin V, a protein with high affinity for PS. Therefore, AnnV conjugated to APC fluorochrome is designed for early detection of apoptosis and in conjunction with membrane permeability dye, as PI, allows to identify live, apoptotic and late apoptotic/secondary necrotic cells. Thus, live cells are both AnnV and PI negative (AnnV−/PI−) (Q1), whereas the early apoptotic cells are AnnV positive and exclude PI due to intact membranes (AnnV+/PI−) (Q2). In contrast, the double AnnV and PI positivity (AnnV+/PI+) and AnnV−/PI+ condition identify the cell population in the late apoptotic/secondary necrotic stage (Q3 and Q4, respectively). Results are representative of two independent experiments

The cytofluorimetric analysis revealed a significant increase in the percentage of total apoptotic cells in the 48-h-long treatment (*p* = 0.012) (Fig. 2); a similar trend is observed in the 24-h-long treatment, even if not statistically significant. No significant effect in the percentage of necrosis was observed. Overall, the results demonstrated that the TXA treatment causes a strong decrease in the percentage of live cells by activating the induction of apoptosis.

**Fig. 3:**
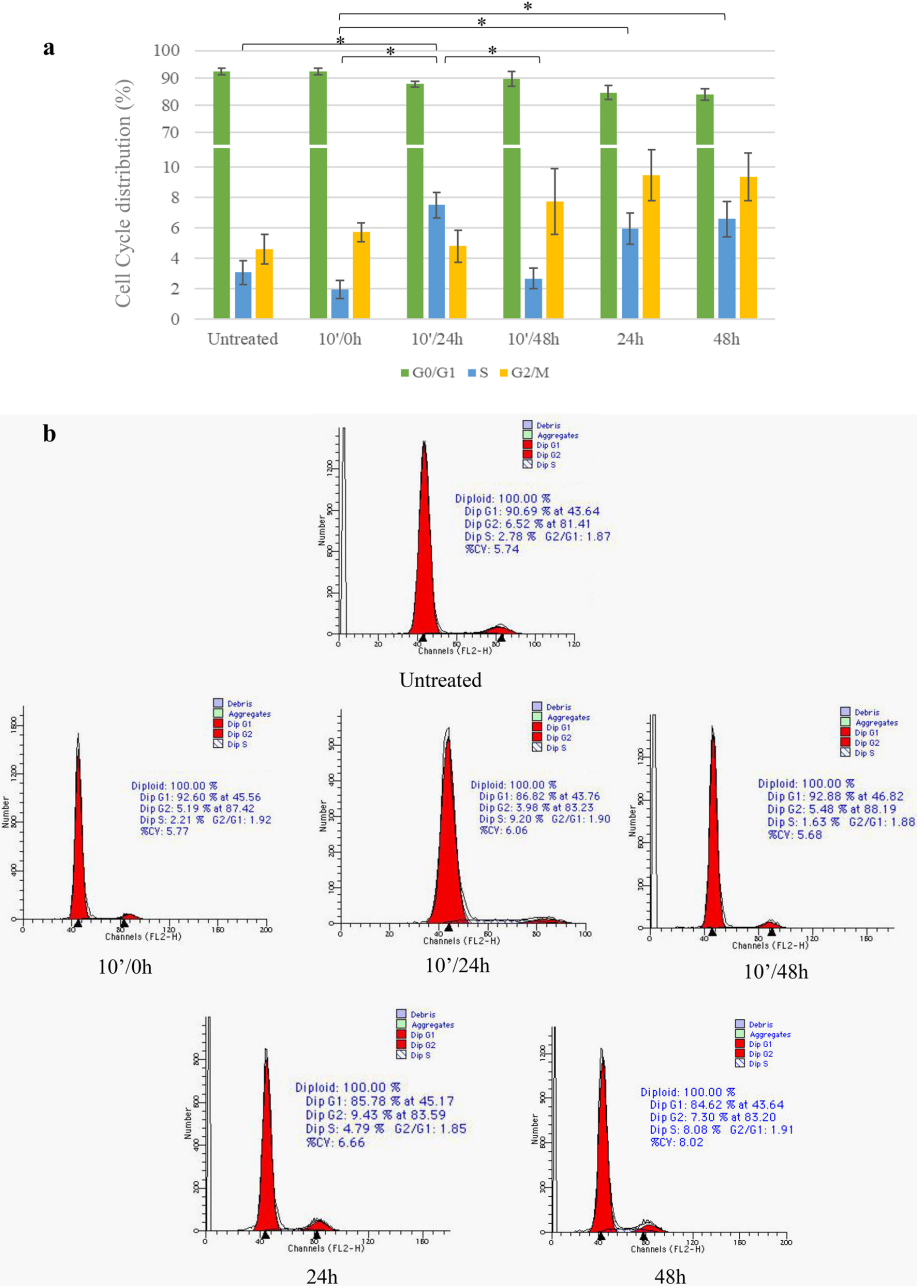
Effect of TXA treatment (100 mg/ml) on chondrocyte cell cycle distribution (%). **a** The graph displays the same data as in Table 3. Note that, for better visualization, G0/G1 bars have been interrupted between 11 and 65%. The results are expressed as mean percent ± SEM of three independent experiments. (*p ≤ 0.05 for Tukey’s test). **b** Flow cytometric charts refer to the most representative experiment among the three performed

To further investigate the mechanism underlying the in vitro biological effects of TXA, its ability to affect the cell cycle distribution has been also assessed. The results from this analysis (Table 3 and Fig. 3) showed that TXA administered over 10 min did not cause any significant alteration in the cell cycle distribution. However, when the cells were incubated for 24 and 48 h, the number of cells in the G0/G1 phase in the cell cycle decreased when compared to control and to 10-min-long treatments. The reduction in the cell population in the G0/G1 phase was associated with a modest increase in the G2/M phase fraction and a marked and significant increase of cells in S at 24 h as well as 48 h, when compared with the 10-min-long treatments. Interestingly, when the TXA was eliminated after 10 min by washing and the cells were incubated with fresh complete medium for 24 h, the cell population in the G0/G1 phase increased with a concomitant reduction of cells in G2/M phases, restoring a biological condition similar to untreated, although the percentage of cells in the S phase at 10′/24 h still did not reach the level of control.

**Table 3:** Effect of TXA treatment (100 mg/ml) on chondrocyte cell cycle distribution (%)

	Moments					
	Untreated	10′/0 h	10′/24 h	10′/48 h	24 h	48 h
Cell cycle phase						
G0/G1	92.35 ± 1.31	92.36 ± 1.15	87.71 ± 1.01	89.61 ± 2.82	84.61 ± 2.6	83.93 ± 2.2
S	3.06 ± 0.79	1.94 ± 0.58	7.50 ± 0.84	2.66 ± 0.66	5.93 ± 1.02	6.58 ± 1.17
G2/M	4.59 ± 0.99	5.71 ± 0.62	4.79 ± 1.04	7.73 ± 2.18	9.45 ± 1.68	9.38 ± 1.57

The results are expressed as mean percent ± SEM of three independent experiments

## Discussion

Tranexamic acid acts as an antifibrinolytic agent binding to plasminogen, preventing the formation of plasmin and, thus, resulting in the inhibition of fibrin degradation [11]. Numerous recent studies have shown the efficacy of intra-articular TXA in controlling perioperative blood loss [12–14] during total joint arthroplasties (in which the removal of the entire articular cartilage is executed) at concentrations ranging between 20 and 100 mg/ml. Conversely, a controversy exists regarding its safe administration in case of bleeding during arthroscopies, joint ligament reconstructions and hemiarthroplasties. At the same time, hemarthrosis, inducing the destabilization of joint homeostasis, may have a detrimental effect on chondrocytes and their viability [15].

With the aim of extending the use of TXA to the aforementioned minimally invasive surgeries, the possible risks of systemic administration being known [16], the present study was designed to evaluate in vitro, in human chondrocytes, whether pharmacological cytotoxicity occurs and whether it is related to the concentration and time of exposure. To the best of our knowledge, only few studies have investigated the same issue; however, they were performed on non-human samples or, if conducted on human chondrocytes, with low TXA doses and short exposure times [10, 17, 18]. This is the first study to evaluate the effect of TXA, in vitro, on human chondrocytes at concentrations higher than 20 mg/ml combined to different times of exposure.

A major problem in in vitro culture of mammalian articular chondrocytes is the process of dedifferentiation, characterized by loss of morphology as well as of functional features. Such dedifferentiation is promoted in culture cells at low density and with repeated passaging [19, 20]. For these reasons, all experiments were carried out on passage 1 chondrocytes, so as to minimize the extent of dedifferentiation over subcultures.

To mimic the composition of the most commonly used clinical formulation, i.e., the vial, we used TXA dissolved in WFI; in parallel, aware that water alone could itself have a cytotoxic effect, we conducted experiments using the same volumes of water (without TXA) as in the previous experiment. Furthermore, to work with a more technically suitable solution for cell cultures, the same experiment was also conducted by dissolving TXA in complete cell medium.

In the literature, effective dosing for topical TXA ranges from 250 mg to 3 g (corresponding to 15–100 mg/ml concentration) [21–23]; thus, we commenced to study the effect on cell viability at 20, 50, 70 and 100 mg/ml, maintaining TXA in culture for 24 and 48 h. To best mimic what theoretically occurs in vivo during surgery (soaking TXA locally just for few minutes and then carrying out the irrigation/aspiration of the solution) [6, 22], we assessed also the effect by limiting the exposure time to 10 min and then evaluating cell viability at 0 (immediately), 24 and 48 h from this short application.

Statistical significance (*p* = 0.020) was obtained from the overall interaction between factors (all types of treatment, all moments and all concentrations).

The reason for the observed lower viability in cells treated 10′ with WFI and W-TXA compared to M-TXA could be explained, in vitro, as resulting from the effect produced by the vehicle (the water): probably, since the osmotic effect, water caused the cell death that affected cell viability measurements; this explanation could justify the toxic effect of W-TXA compared to M-TXA.

Successively, instead, observing the dose-dependent response for all treatments after 24 and 48 h of exposure, we could hypothesize that the former highlighted effects on cell viability are only partially attributable to a solvent effect of water; in fact, the effect of both W-TXA and M-TXA is more severe than that of the WFI itself, leading to the conclusion that the TXA has an intrinsic pharmacological cytotoxicity which affects cell viability more than the osmotic effect itself.

The analysis reported in Table 3 led us to identify the critical role of concentration and time of exposure in cytotoxicity: in fact, the effect of the drug (both M-TXA and W-TXA) at 20 mg/ml has not shown any significant change in cell viability, regardless of time of exposure, whereas 50 and 70 mg/ml may be considered safe for both M-TXA and W-TXA, only if washes are performed. The concentration of 100 mg/ml is safe only for M-TXA and if washes are performed.

Our results align with Tuttle et al. [10], who performed a study to understand the in vitro effects of TXA on bovine and murine chondrocytes, reporting that an extended exposure to TXA at high concentrations is cytotoxic to cartilage, while chondrocyte viability was unaffected at lower concentrations. Also, the studies by Ambra et al. (on porcine cartilage explants) [17] and Sitek et al. [24] support our findings that a reduced TXA exposure and at a low concentration could be safely used. Parker et al. [18] the observed cytotoxic effects with concentrations above 20 mg/ml of TXA when treatments were prolonged up to 12 h. However, these studies did not control for increased time of TXA exposure, higher concentrations or the possibility to wash away the TXA after short treatments.

In light of this, our findings widen the horizons, focusing on time of exposure as the most critical factor in TXA toxicity. In fact, limiting the exposure of M-TXA only for a few minutes can be considered safe even at the highest concentration studied, whereas for W-TXA, the highest safe concentration is 70 mg/ml. Conversely, an increased exposure time to 24 and 48 h without a wash may damage cartilage due to the direct cytotoxicity of the drug even at 50–70 mg/ml.

Once the cytotoxic effect was tested, we elucidated the mechanism by which cell death occurred. To avoid a possible “water effect” of the solvent, we only analyzed the toxic effect on apoptosis/necrosis and cell cycle distribution of M-TXA at the highest concentration. Labeling with AnnV/PI showed a significant increase of apoptotic cells after 48 h of treatment and a concomitant strong reduction of live cells compared to the controls. No significant effect, instead, was observed for necrosis.

Moreover, the results obtained by cell cycle analysis by flow cytometry highlighted a reduction in the cell population in the G0/G1 phase at 24 h and 48 h, which was followed by an accumulation of chondrocytes in the S and G2/M phases. Interestingly, no significant difference was observed between untreated and 10′-treated cells, suggesting that the washing is a beneficial step to restore the basal condition and to prevent the cytotoxic effect of the drug.

We are aware that this study is limited by the use of chondrocytes cultures obtained from osteoarthritic knee that has undergone a TKR, and that the viability could be influenced by their native condition and/or age [25, 26]; it has been highlighted, in fact, that healthy/OA chondrocytes differ for some biological features [25] and that, similarly, articular chondrocytes exhibit an age-related modulation of some biological processes related to proliferative and synthetic capacity or the ability to produce pro-inflammatory mediators and matrix-degrading enzymes [26]. Our data do not give suggestions about the possible TXA mechanism of action, so we cannot hypothesize if it acts through an age-related or physiological state-related mechanism and, consequently, if our experimental observations can be extended to all the chondrocytes’ biological conditions.

We also would like to stress out that the study was performed using the most relatively intact cartilage from samples, that the comparison of treated and untreated cells should be a reliable internal control and, ultimately, that several studies not specifically concerning osteoarthritic disease had already used osteoarthritic chondrocytes as cell culture model [27–29]. Moreover, the laboratory effect of water-dissolved solutions (we hypothesized an osmotic damage) is not completely clear and we will further investigate.

We wondered if the systemic TXA administration could have similar effects on articular chondrocytes. Usually, after a TXA intravenous administration of typical doses of 10–15 mg/kg body weight, the concentration in plasma reaches 18, 10 and 5 mg/ml, respectively, after 1, 3 and 5 h [30]; it is also known that TXA intravenously administered diffuses rapidly into the synovial fluid until its concentration equals that in the serum [30, 31]. In light of this, we could assume that the toxic effect of intravenous administration on articular chondrocytes should be lower than the topical one.

This study also opens a debate on the TXA molecular mechanism of cell death induction; it has been recently demonstrated, however, that TXA 100 mg/ml may partially act trough a caspase-3-dependent apoptotic mechanism, not only in chondrocyte, but also in other cell types (such as synoviocytes and tenocytes) [32] suggesting a strong involvement of apoptosis molecular machinery.

## Conclusion

The presented data suggest that caution in the time of TXA soaking is critical and making a wash after few minutes is mandatory to avoid cytotoxicity; a prolonged exposure could be a cause of cartilage damage also at TXA concentrations lower than 100 mg/ml. Since the most useful clinical formulations for TXA is the vial (that resembles the W-TXA formulation), this study suggests clinicians to show caution when using topical TXA administration (concentrations of 70 mg/ml or lower would be advisable when a procedure with preservation of native cartilage is performed).

However, before topical TXA administration could be recommended for routine clinical practice. These data have to be supported by future in vivo studies considering pharmacological features, such as drug clearance and tissue distribution that characterize the in vivo administration; human clinical trials are also required.

## Abbreviations

TXA: tranexamic acid
THR: total hip replacement
TKR: total knee replacement
W-TXA: water-dissolved tranexamic acid
M-TXA: medium-dissolved tranexamic acid
WFI: water for injections
OD: optical density
HSD: honestly significant difference
PS: phosphatidylserine
SEM: standard error of mean

## References

[1] Sukeik M, Alshryda S, Haddad FS, Mason JM (2011). Systematic review and meta-analysis of the use of tranexamic acid in total hip replacement. J Bone Joint Surg Br.

[2] Stowers MDJ, Aoina J, Vane A, Poutawera V, Hill AG, Munro JT (2017). Tranexamic acid in knee surgery study—a multicentered, randomized, controlled trial. J Arthroplasty.

[3] Henry DA, Carless PA, Moxey AJ, O’Connell D, Stokes BJ, Fergusson DA (2011). Anti-fibrinolytic use for minimising perioperative allogeneic blood transfusion (review). Cochrane Database Syst Rev.

[4] Murkin JM, Falter F, Granton J, Young B, Burt C, Chu M (2010). High-dose tranexamic acid is associated with non-ischemic clinical seizures in cardiac surgical patients. Anesth Analg.

[5] Panteli M, Papakostidis C, Dahabreh Z, Giannoudis PV (2013). Topical tranexamic acid in total knee replacement: a systematic review and meta-analysis. Knee.

[6] Georgiadis AG, Muh SJ, Silverton CD, Weir RM, Laker MW (2013). A prospective double-blind placebo-controlled trial of topical tranexamic acid in total knee arthroplasty. J Arthroplasty.

[7] Jang B, Kao M, Bohm MT, Harris IA, Chen DB, MacDessi SJ (2014). Intra-articular injection of tranexamic acid to reduce blood loss after total knee arthroplasty. J Orthop Surg.

[8] Tzatzairis TK, Drosos GI, Kotsios SE, Ververidis AN, Vogiatzaki TD, Kazakos KI (2016). Intravenous vs topical tranexamic acid in total knee arthroplasty without Tourniquet application: a randomized controlled study. J Arthroplasty.

[9] Telhag H (1973). Effect of tranexamic acid (CyklokapronR) on the synthesis of chondroitin sulphate and the content of hexosamine in the same fraction on normal and degenerated joint cartilage in the rabbit. Acta Orthop Scand.

[10] Tuttle JR, Feltman PR, Ritterman SA, Ehrlich MG (2015). Effects of tranexamic acid cytotoxicity on in vitro chondrocytes. Am J Orthop.

[11] Nilsson IM (1980). Clinical pharmacology of aminocaproic and tranexamic acids. J Clin Pathol Suppl.

[12] Wong J, Abrishami A, El Beheiry H, Mahomed NN, Roderick Davey J, Gandhi R (2010). Topical application of tranexamic acid reduces postoperative blood loss in total knee arthroplasty: a randomized, controlled trial. J Bone Joint Surg Am.

[13] Wang J, Wang Q, Zhang X, Wang Q (2017). Intra-articular application is more effective than intravenous application of tranexamic acid in total knee arthroplasty: a prospective randomized controlled trial. J Arthroplasty.

[14] Sun X, Dong Q, Zhang YG (2016). Intravenous versus topical tranexamic acid in primary total hip replacement: a systemic review and meta-analysis. Int J Surg.

[15] Hooiveld M, Roosendaal G, Wenting M, van den Berg M, Bijlsma J, Lafeber F (2003). Short-term exposure of cartilage to blood results in chondrocyte apoptosis. Am J Pathol.

[16] Patel JN, Spanyer JM, Smith LS, Huang J, Yakkanti MR, Malkani AL (2014). Comparison of intravenous versus topical tranexamic acid in total knee arthroplasty: a prospective randomized study. J Arthroplasty.

[17] Ambra LF, de Girolamo L, Niu W, Phan A, Spector M, Gomoll AH (2017). No effect of topical application of tranexamic acid on articular cartilage. Knee Surg Sports Traumatol Arthrosc.

[18] Parker JD, Lim KS, Kieser DC, Woodfield TBF, Hooper GJ (2018). Is tranexamic acid toxic to articular cartilage when administered topically?. Bone Jt J.

[19] Watt FM (1988). Effect of seeding density on stability of the differentiated phenotype of pig articular chondrocytes in culture. J Cell Sci.

[20] Tew SR, Murdoch AD, Rauchenberg RP, Hardingham TE (2008). Cellular methods in cartilage research: primary human chondrocytes in culture and chondrogenesis in human bone marrow stem cells. Methods.

[21] Alshryda S, Sukeik M, Sarda P, Blenkinsopp J, Haddad FS, Mason JM (2014). A systematic review and meta-analysis of the topical administration of tranexamic acid in total hip and knee replacement. Bone Jt J.

[22] Kim TK, Chang CB, Koh IJ (2014). Practical issues for the use of tranexamic acid in total knee arthroplasty: a systematic review. Knee Surg Sports Traumatol Arthrosc.

[23] Sabatini L, Atzori F (2015). Topical intra-articular and intravenous tranexamic acid to reduce blood loss in total knee arthroplasty. Ann Transl Med.

[24] Sitek P, Wysocka-Wycisk A, Kępski F, Król D, Bursig H, Dyląg S (2013). PRP-fibrinogen gel-like chondrocyte carrier stabilized by TXA-preliminary study. Cell Tissue Bank.

[25] Yang KG, Saris DB, Geuze RE, van Rijen MH, van der Helm YJ, Verbout AJ (2006). Altered in vitro chondrogenic properties of chondrocytes harvested from unaffected cartilage in osteoarthritic joints. Osteoarthritis Cartilage.

[26] Loeser RF (2009). Aging and osteoarthritis: the role of chondrocyte senescence and aging changes in the cartilage matrix. Osteoarthritis Cartilage.

[27] Kim TW, Lee MC, Bae HC, Han HS (2018). Direct coculture of human chondrocytes and synovium derived stem cells enhances in vitro chondrogenesis. Cell J.

[28] Jeyakumar V, Niculescu-Morzsa E, Bauer C, Lacza Z, Nehrer S (2017). Platelet rich plasma supports proliferation and redifferentiation of chondrocytes during in vitro expansion. Front Bioeng Biotechnol.

[29] Secretan C, Bagnall KM, Jomha NM (2010). Effects of introducing cultured human chondrocytes into a human articular cartilage explant model. Cell Tissue Res.

[30] Astedt B (1987). Clinical pharmacology of tranexamic acid. Scand J Gastroenterol Suppl.

[31] Chen JY, Chia SL, Lo NN, Yeo SJ (2015). Intra-articular versus intravenous tranexamic acid in primary total knee replacement. Ann Transl Med.

[32] McLean M, McCall K, Smith IDM, Blyth M, Kitson SM, Crowe LAN (2019). Tranexamic acid toxicity in human periarticular tissues. Bone Jt Res.

